# Peptide Sequencing Directly on Solid Surfaces Using MALDI Mass Spectrometry

**DOI:** 10.1038/s41598-017-18105-3

**Published:** 2017-12-19

**Authors:** Zhan-Gong Zhao, Lalaine Anne Cordovez, Stephen Albert Johnston, Neal Woodbury

**Affiliations:** Biodesign Institute Center for Innovations in Medicine, Arizona State University, Tempe, AZ 85287 United States of America

## Abstract

There are an increasing variety of applications in which peptides are both synthesized and used attached to solid surfaces. This has created a need for high throughput sequence analysis directly on surfaces. However, common sequencing approaches that can be adapted to surface bound peptides lack the throughput often needed in library-based applications. Here we describe a simple approach for sequence analysis directly on solid surfaces that is both high speed and high throughput, utilizing equipment available in most protein analysis facilities. In this approach, surface bound peptides, selectively labeled at their N-termini with a positive charge-bearing group, are subjected to controlled degradation in ammonia gas, resulting in a set of fragments differing by a single amino acid that remain spatially confined on the surface they were bound to. These fragments can then be analyzed by MALDI mass spectrometry, and the peptide sequences read directly from the resulting spectra.

## Introduction

Solid-phase peptide synthesis, pioneered by Bruce Merrifield^1^, has allowed rapid, low-cost, and accurate synthesis of peptides. Traditionally, only a small number of peptides were synthesized at once. As a result, there was no need for high throughput quality control methods including sequence analysis. With the development of high throughput peptide synthesis and, in particular, with peptides increasingly being used while attached to solid surfaces, peptide chemists are now frequently dealing with numbers of peptides from thousands to millions in an experiment. Peptide sequencing becomes increasingly essential in quality evaluation during synthesis or in lead identification and confirmation from combinatorial libraries. For example, on-bead screening of a “one-bead one-peptide” library^2^ is among the most popular approaches for discovery of specific peptide ligands, enzyme substrates^3,4^ or inhibitors^5,6^. Hundreds of peptide leads are obtained from a single assay, and each lead needs to be characterized via sequencing. Peptide arrays on paper^7^, glass^8^, and silicon wafer^9^ surfaces have been used similarly for the discovery of ligands against proteins, virus particles^10^ and bacteria^11^ and for the measurement of antibody profiles used in disease diagnosis^9,12–15^. These arrays are usually synthesized *in situ* and, by necessity, used without either purification or proper quality evaluation such as direct sequence analysis. The ability to perform sequence analysis on specific peptides in an array could be used in lot-based quality control and to evaluate the extent of side reactions during synthesis and processing (e.g., incomplete coupling, premature alpha-amino de-protection, and side chain modifications). There is thus a need for a high throughput approach to analyzing peptides *in-situ* on solid surface that can be implemented with commonly available instrumentation.

Common methods of peptide sequencing at present include automated Edman^16,17^ degradation and Tandem mass spectrometry (MS/MS)^18,19^; both methods have limited throughput for various reasons. Edman degradation can be used in direct sequencing of peptides immobilized on solid resin beads, but the process is slow and requires multi-cycles of chemical transformations and HPLC analyses. Peptide sequencing using tandem MS is faster, but this method requires the peptides to be released into solution before analysis, and uses sophisticated instrumentation requiring specialized facilities and personnel^19,20^. Other methods, such as ladder sequencing^21–23^, have been developed for sequencing peptides in solution phase; without further development, these methods are not applicable to direct determination of peptide sequences attached to surfaces.

**Figure 1:**
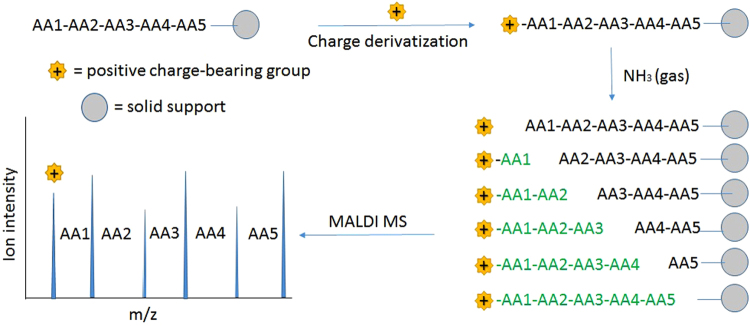
Process of mass spectroscopic analysis of peptide ladders generated in gas phase ammonia. A peptide (with amino acids AA1 through AA5 in the scheme) is synthesized on a solid support (represented as a gray circle). The N-terminal amine is labeled by a group with a permanent positive charge. This is subjected to high pressure ammonia gas which randomly cleaves peptide bonds, resulting in a series of N-terminal labeled fragments. Generally, only the charge-labeled fragments give a strong signal in the mass spectrum. Upon MALDI mass spectrometry, one sees a series of peaks that correspond to masses separated by single amino acid molecular weights.

Here, a simple approach for peptide sequencing is presented that involves controlled chemical degradation in the gas phase (Fig. 1). A peptide, attached to a solid support such as a resin bead, is first derivatized at its N-terminus with a positive charge-bearing group. The peptide is then subjected to controlled chemical degradation at high pressure (~100 PSI) ammonia gas. Hundreds of individual beads can be physically immobilized at the bottom of a petri dish and treated in one experiment. A family of N-terminal labeled peptide fragments results from the treatment, each differing from the next by a single amino acid. Because the cleavage is performed in the gas phase, the cleavage products remain in the same beads as the peptide that gave rise to them. Therefore, peptides fragments generated on individual beads can be extracted into a small volume (~1uL) of matrix solution and analyzed with a conventional Matrix-Assisted Laser Desorption/Ionization (MALDI) mass spectrometer. The potential for extending this approach to sequencing peptide features in microarrays will be discussed.

## Results

### Charge Derivatization

Derivatization of peptides with fixed charges has been shown to greatly increase detection sensitivity, simplify fragmentation patterns and, thus, facilitate interpretation of mass spectra in MS/MS based *de novo* peptide sequencing^24,25^. The approach described here does not involve fragmentation in the process of mass spectra acquisition. But we have observed that attaching a fixed positive charge to the N termini of the peptides (Supplementary Fig. 1) indeed helped the detection of peptide fragments, possibly by increasing detection sensitivity or by facilitating peptide fragmentation in ammonia gas. Without labeling, no peptide bond breakage was observed after a short peptide was treated in high pressure of ammonia gas (Supplementary Fig. 2). This indicated that peptide bonds are generally quite stable in ammonia gas and any low level of peptide bond cleavage results in fragments with too low an abundance to be detected in the mass spectrum. However, when labeled at its N-terminus with a positive charge-bearing group, the same peptide gave rise to detectable fragments from cleavages at each amide bond (peptide bond). The MALDI mass spectrum of these fragments correctly corresponded to the sequence of the peptide (Supplementary Fig. 3). A phosphonium group, N-Tris(2,4,6-trimethoxypheyl)phosphonium (TMPP), was selected as the positive charge-bearing group. It has the advantage of not only being permanently charged, but it also greatly increases the molecular weight of the small peptide fragments, avoiding overlap in the spectrum with the low molecular weight ions that arise from the matrix.

**Figure 2:**
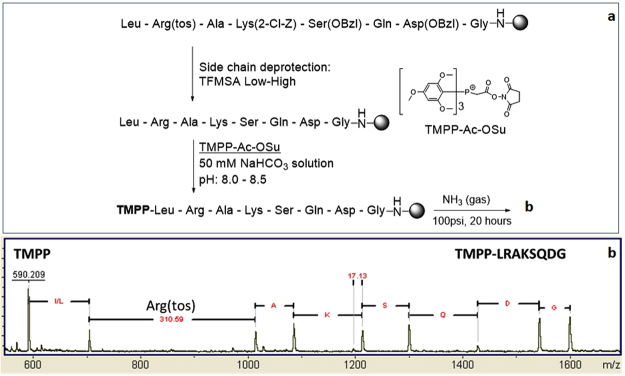
Selective derivatization of a peptide at its N-terminus for sequence analysis. **(a**) An 8 mer peptide was synthesized using BOC chemistry; the peptide’s side chains were deprotected by a conventional TMFSA Low-High procedure before it was derivatized with TMPP-Ac-OSu under controlled pH (8.0–8.5). (**b**) A few beads of the derivatized peptide were treated in ammonia gas and analyzed by MALDI mass spectrometry. The spectrum was not calibrated.

Depending on the purpose of an analysis, different approaches may be used in the labelling of a surface-immobilized peptide. The attachment of TMPP in two steps as shown in Supplementary Fig. 1 is easily applied in situations where quality control of the synthesis is the primary objective. However, peptide sequencing is frequently needed for identification and characterization of unknown peptide leads selected from a ligand discovery assay (e.g., a bead library) where the side chain protection groups of peptides are previously removed. Thus, a method of selectively attaching TMPP to the N terminus of a peptide in presence of other basic residues such as Lys, His, Arg is necessary. Wetzel *et al*. presented a selective procedure for modification of N-terminal amino groups by exploiting differences between the pKa of the N-terminal alpha amino group (~8.0) and the pKa of the more basic amino acid side chains (~10.5 for Lys, ~12.0 for Arg)^26^. Others have used the same approach for charge derivatization of peptides selectively at their N-termini for *de novo* analysis by MS/MS^25,27,28^. To test this selective derivatization approach on surface bound peptides, we synthesized an 8-mer peptide that includes a lysine residue and an arginine residue (Fig. 2a). The peptide was synthesized using BOC chemistry without a cleavable linker. After its side chains were removed by using a modified TFMSA procedure^29^, the N-terminus of this peptide was derivatized with N-Succinimidyl [tris(2,4,6-trimethoxyphenyl)phosphonio]acetate bromide (TMPP-Ac-OSu, commercially available from Sigma) under controlled pH (0.1 M NaHCO_3_, pH = 8.0–8.5). A few beads of this labeled peptide were then treated with ammonia gas, and a small volume (1uL) of matrix solution was added to extract the peptide fragments from two of the beads for analysis by MALDI mass spectrometry. The MALDI mass spectrum (Fig. 2b) showed that only the N-terminus was derivatized as desired and gave the correct sequence of the peptide. Interestingly, the spectrum also indicated that the side chain deprotection condition is not adequate to remove side chain protecting groups of arginine and thus showed the power of this approach in detecting problems in synthesis.

### Peptide degradation in ammonia gas

**Figure 3:**
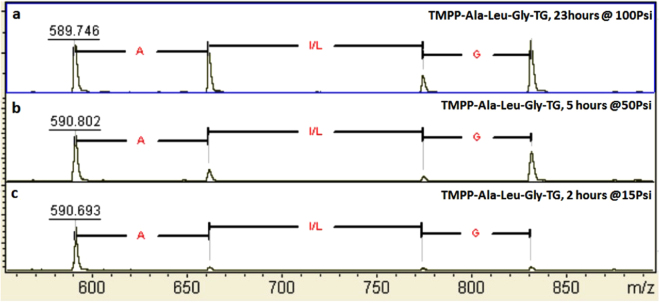
MALDI mass spectra of TMPP labeled trimer peptide, TMPP-Ala-Leu-Gly-TG (TG = TentaGel beads). (**a**) The peptide was treated in ammonia gas for 23 hours at 100 Psi. (**b**) 5 hours at 50Psi. (**c**) 2 hours at 15PSI. The spectra were not calibrated.

For practical application of our sequencing approach, the appropriate conditions of ammonia treatment in terms of time and pressure were determined, so that a set of peptide fragments is generated yielding a complete peptide sequence. We have observed in Fig. 2 and also in Supplementary Fig. 2 that peptide bonds are quite stable, and over-degradation is unlikely to be a concern even in high ammonia pressure. This allowed us to perform the process as a function of time and pressure over wide range. A trimer peptide was synthesized on TentaGel (TG) beads and labeled with TMPP: TMPP-A-L-G-TG. The peptide was then treated in ammonia gas for different periods of time and at different pressures. Figure 3 shows the MALDI mass spectra *vs*. time and pressure, indicating that peptide bonds are cleaved to a significant extent even with short treatment and at very low ammonia pressure (Fig. 3c). With longer time treatment at high pressure (Fig. 3a), three of the four peptide bonds were close to equal in their lability to degradation in ammonia gas. However, differences in the lability of peptide bonds to ammonia gas were observed in all three conditions in Fig. 3. Realizing that the liability of peptide bonds may be sequence dependent, we selected the following sequences as test cases: TMPP-LXSQDG-TG with X = H, K, N, E, P, R, T, W, Y, I. the peptides were synthesized on TentaGel (TG) beads by Fmoc chemistry and without a cleavable linker. With these peptides, the effects of each amino acid, X, were determined for the cleavage of the L-X bond at the N-terminal side and for the cleavage of the X-S bond on the C-terminal side of the amino acid in question.

**Figure 4:**
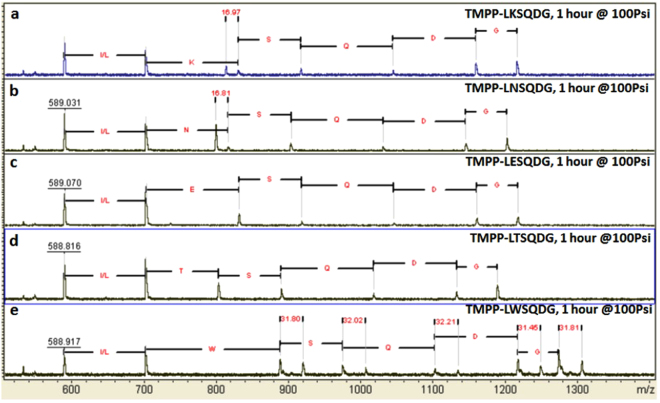
MALDI mass spectra of TMPP labeled six-mer peptides, TMPP-L-X-S-Q-D-G-TG. TG = TentaGel; (**a**) X = K; (**b**) X = N; (**c**) X = E; (**d**) X = T; (**e**) X = W. The mass spectra were not calibrated.

**Figure 5:**
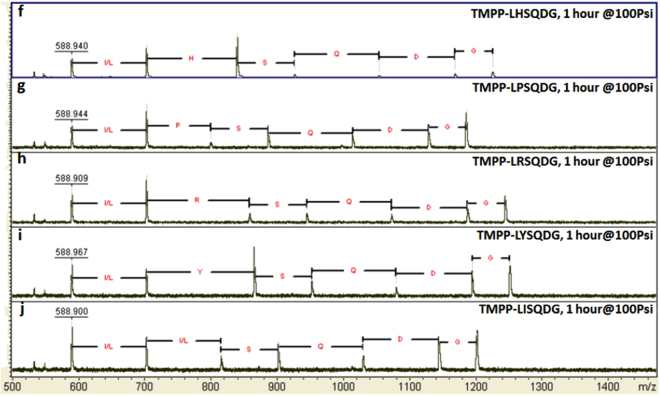
MALDI mass spectra of TMPP labeled six-mer peptides, TMPP-L-X-S-Q-D-G-TG. TG = TentaGel; (**f**) X = H; (**g**) X = P; (**h**) X = R; (**i**) X = Y; (**j**) X = I. The mass spectra were not calibrated.

Figures 4 and 5 show the mass spectra of the ten sequences. The relative intensities of molecular ions, TMPP and TMPP-L, are the highest and almost constant in all ten spectra, indicating that an amino acid does not greatly affect lability of peptide bonds at its N-terminal side. The intensities of molecular ions due to cleavage on the C-terminal side, however, are to some extent affected. For example, the intensities of molecular ions, TMPP-L-X, vary with the amino acid in the X position and are greater when X = N, T, W, H, and Y, and relatively smaller when X = K, E, P, R, and I. In addition, the intensities of molecular ions, TMPP-L-X-S and TMPP-L-X-S-Q, also vary with the amino acid at the position of X, indicating that the effect of an amino acid on peptide bonds at its C-terminal side goes beyond one or two amino acids. For X = H, the relative intensity of TMPP-L-H is the greatest in the spectrum, while the relative intensities of TMPP-L-H-S and TMPP-L-H-S-Q are the lowest. To the contrary, when X = P, the intensity of TMPP-L-P is the lowest in the spectrum, cleavage of the next two peptides bonds results in a TMPP-L-P-S and TMPP-L-P-S-Q with relatively much higher intensities. Another interesting observation is that the intensity of molecular ion, TMPP-L-T (Fig. 4d), is larger than that of TMPP-R-Y-T as seen in Supplementary Fig. 3, also indicating that the lability of a peptide bond in ammonia gas may be affected by neighboring amino acids in the peptide. At present, the effects of amino acids in a peptide on their surrounding amide bonds and the mechanism involved remain to be explored. However, cleavage at all possible positions was detectable, even using a relatively low pressure of ammonia gas over short time period, implying that the approach is robust. The axis scales in Figs 4 and 5 were reset to magnify the smaller peaks and presented as Supplementary Figs 4 and 5.

### Amino acid annotation for sequence readout

For sequencing purposes, the analysis primarily consists of determining the molecular weight differences between adjacent molecular ions in the spectrum; therefore, it was not necessary to calibrate the MALDI mass spectra for accurate absolute masses. To eliminate analytical subjectivity, we performed amino acid annotation of adjacent peaks by using a software package provided by Bruker: Daltonic flexAnalysis 3.0. Using MS based sequencing methods, it is not possible to discriminate between amino acids, I and L, since their molecular weights are identical. It is also difficult to tell K and Q apart as their molecular weights differ only by 36 mDa. Depending on spectral resolution, the 1 Da difference between D and N, or E and Q, can also be challenging. However, in some cases either fortuitous or purposeful modification of amino acids can render them easier to distinguish. In Fig. 2b, there is a peak 17 Da smaller than the peak annotated to Lys. As also shown in Fig. 4a, this peak most likely corresponds to the deamination of lysine. Interestingly, in all ten spectra of Figs 4 and 5, there is no such a peak accompanying the peak annotated as Q in these spectra, making it possible to easily distinguish between K and Q. On the other hand, N does show a deamination peak and this distinguishes it easily from D under the conditions of this preparation (Fig. 4b). Note that, in Fig. 4e, there is a +32 peak found for every peptide ion that has a W residue. Most likely this peak is due to oxidation of tryptophan to N-formylkynurenine during side chain deprotection under acidic conditions.

**Figure 6:**
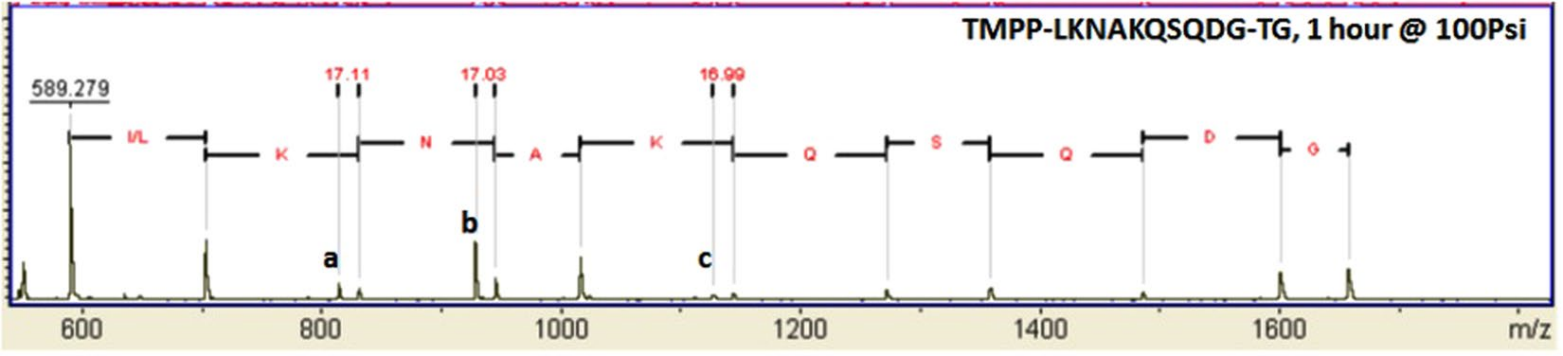
Deamination of Lysine and Asparagine in ammonia gas. Deamination (−17 Da) peaks are labeled as (**a)** for Lys (K), (**b)** for Asn (N) and (**c**) for Lys (K). No deamination is observed for the two Gln (Q) residues. The MALDI mass spectrum was not calibrated. The peptide was synthesized on Tenta Gel (TG) beads without a cleavable linker and treated in ammonia for 1 hour at 100 PSI.

To further confirm the deamination of K and N in Fig. 4, we synthesized a longer peptide, TMPP-LKNAKQSQDG, on TentaGel (TG) beads without a cleavable linker. With this peptide we wanted to determine whether deamination of both lysine residues takes place if they are in the same peptide and also to see if both Q residues are free from deamination as was the case in Figs 4 and 5. The resulting MALDI mass spectrum (Fig. 6) shows that both lysine (K) residues in the same peptide were partially deaminated, while no clear indication of deamination was observed for the two glutamine (Q) residues in the same peptide, allowing one to distinguish K and Q in this peptide. In Fig. 6, although all of the required ionized fragments are present and easily assigned, several have low intensities. Supplementary Fig. 6 gives a magnified version of this spectrum, showing the weaker signals more clearly. As was observed in Figs 4b and 6 also shows significant deamination of Asn (N), thus distinguishing it from Asp (D).

## Discussion

Here we describe a sequence analysis approach that is based on a partial peptide degradation in ammonia gas and is particularly robust for peptide sequence analysis directly on solid surface. We first demonstrated that peptide bonds are cleavable in ammonia gas, labeling of peptides at their N-termini with TMPP allows sequence determination by MALDI mass spectrometry. We then showed that the cleavage of the peptides bonds are sequence dependent and could be controlled by varying exposure time and pressure of ammonia gas. Finally, we demonstrated that side-chain modification in ammonia gas could be used in sequence determination to distinguish between amino acids with the same or very similar molecular weights.

As the most essential step in this approach, degradation of peptides must be controlled to an appropriate extent for correct readout of the sequences; both insufficient- and over-degradation will reduce the number of fragments required for a complete sequence readout. Our results show that peptide degradation in ammonia gas is easily controlled in this regard. First, unlike peptide hydrolysis in aqueous solutions^30–32^, peptide (amide) bonds are relatively stable in ammonia gas so that cleavage is rare and, therefore, it is very unlikely that a peptide would be over-degraded in ammonia gas. Second, by attaching a positive charge-bearing group to the N terminus of each peptide, the resulting set of peptide fragments, even though low in abundance, are easily detectable in the MALDI mass spectra after a short period of treatment in ammonia gas (Fig. 3). Depending on the specific needs of the analysis, the peptide can be derivatized at its N-terminus either by a two-step process before side chain deprotection, or by a pH controlled one-step process after the peptide is deprotected.

As with other MS based sequencing approaches, unambiguously distinguishing between certain amino acids with identical or similar molecular weights (I/L, K/Q, Q/E, and N/D) presents a challenge, particularly if one hopes to use the method with lower resolution, less expensive, more available mass spectrometers. This problem can be alleviated at least for K/Q and N/D by considering fortuitous or purposeful modifications of these amino acids, as described above. It is not clear what the mechanism is for deamination of K and N in this process nor is it clear why N gives a deamination product while Q does not. However, the ability to discriminate between K and Q, as well as N and D, without having to perform specific modification chemistry is a useful aspect of this approach.

In the work reported here, we focused on developing the sequencing approach for bead-based libraries. This is an important application. At the speed of this method, Peptides on individual beads could be sequenced in a relatively short time period, making it possible to perform, for example, a binding selection procedure and then sequence the top hundreds of binders. However, there is no inherent reason why this approach cannot be extended to other surface based peptide applications as well, and with the increase in the variety, size and application of *in situ* synthesized peptide arrays this has become increasingly important for quality control. We have previously shown that when an array of peptides is synthesized *in situ* on a thermal oxide-coated silicon wafer surfaces, with each peptide having an ammonia-labile linker at the C-terminus, cleavage from the surface in ammonia gas can be performed and the molecular weight of the peptide in each feature can be determined by MALDI MS imaging^9^. This demonstrates that one can perform ammonia gas exposure to such an array, that peptides free on the surface after such an exposure can be treated with matrix in such a way that excessive diffusion prior to MALDI imaging can be avoided and that there is sufficient peptide in a microarray feature to provide sufficient signal. Sequencing of the peptide arrays would differ from the process in ref.^9^ only in that no base-labile linker would be used and that the level of ammonia gas cleavage would need to be optimized under those conditions.

In summary, the fact that amide bond cleavage in this approach is carried out by gas phase ammonia, without addition of other chemicals to the sample, minimizes the manipulations involved and makes this approach ideal for high speed and high throughput sequence analysis. No cleavable linker is required during synthesis as the peptides are not released, only fragmented *in situ*, for analysis. The positive charge-bearing group added to the N-terminus provides the sensitivity required to detect low abundance fragments needed in the sequencing analysis and serves as an internal reference in MALDI MS analysis. After a small volume of matrix solution is applied, MALDI mass spectra can be acquired with an inexpensive, widely available desktop mass spectrometer in a matter of seconds per peptide. The peptide sequence can be read directly from the mass spectra without the need of sophisticated processing, a process that should be amenable to automation.

## Methods

### TMPP derivatization

All peptides reported here were synthesized on TentaGel amino resin without a cleavable linker, details are described in Supplementary information. Peptides can be derivatized with TMPP (tris(2,4,6-trimethoxyphenyl)phosphonium acetic acid) at their N-terminal either during the synthesis for quality analysis or after the synthesis is complete and side-chain deprotected for lead identification. During the synthesis, TMPP can be attached to the peptides at their N-terminal in two steps as shown in Supplementary Fig. 1; typically, a small sample or resin (~1–5 mg) is removed from the synthesis and washed with Methanol/Dichloromethane in a small column and suspended in a solution of bromoacetic acid (0.1 M), 6-chloro-1-hydroxybenzotriazole (0.1 M), and Diisopropylcarbodiimide (0.1 M) in N-methylpyrrolidinone (0.2 mL) and shaken for 1 hour at room temperature. The resin is then washed with N-methylpyrrolidinone to completely remove unreacted chemicals before being suspended in a solution of tris(2,4,6-trimethoxyphenyl)phosphine (0.1 M) in N-methylpyrrolidinone; the reaction is allowed to proceed at room temperature for 2 hours to ensure the Bromine atom is completely displaced by the phosphine, forming a positive charge-bearing phosphonium at the N terminus of a peptide. The derivatized resin is then ready for ammonia gas degradation with/without side chain deprotection (see Fig. 3).

For selective derivatization of a fully deprotected peptide at its N-terminus in presence of other basic amino acids such as Lysine and Arginine, a small sample of peptide on TentaGel beads in a column (sample size varied from a few hundred beads to a few milligrams depending on experimental purpose) are suspended and shaken in aqueous solution of NaHCO3 (100 mM, pH = 8.0–8.5) for 30 min; after the buffer solution is removed, a solution of TMPP-Ac-Suc (1 mg) in 500uL acetonitrile-100mM NaHCO3 (1:9) is added to the resin sample and shaken for 1 hour at room temperature. The resin sample is then washed with water (5 × 1 min), acetonitrile (3 × 1 min), Methanol (3 × 1 min) and DCM (3 × 1 min). The resin is then dried in *vacuo* before subjected to ammonia treatment.

### Peptide degradation in ammonia gas

A stainless steel gas chamber was designed to hold sample vials of various sizes; a stainless steel cap, equipped with inlet and outlet connectors, was screw-fastened to the top of the chamber to withstand pressures well over 100 PSI. An ammonia tank was linked to the chamber through a polypropylene inlet tubing and a gas pressure gauge, through which the pressure in the chamber can be adjusted; a valve was placed at the outlet and linked to a water trap to absorb ammonia gas coming out of the chamber. The degradation process was initiated by flowing ammonia gas through the system to remove air until no air bubble coming out of the water trap. Ammonia pressure was then increased to 100 PSI after closing the valve on the outlet and the reaction was allowed to proceed undisturbed for 1 hour. After the reaction, resin beads in vials, or individual beads spread across the bottom of a petri dish, were allowed to vent out residual ammonia before MS analysis. A detailed description of the ammonia gas degradation system can be found in Supplementary information 2.

### MS analysis

MALDI mass spectra were all recorded on Bruker Microflex^TM^ spectrometer, which is an entry-level instrument ideal for the non-expert user without specialized training. The spectrometer is equipped with a nitrogen laser (337 nm, 3 ns pulse width, 150 μJ pulse energy) with variable repetition rate, and a microScout ion source with state-of the art pulsed ion extraction. The Bruker’s microScout target, MSP 96 polished steel target plate, was used for all of our analysis. FlexControl^TM^ software module was used for instrument control, all spectra were recorded in linear mode. FlexAnalysis^TM^ were used for all data analysis. As matrix, a saturated solution of α-cyano-4-hydroxycinnamic acid (CHCA) was made in 50% aqueous acetonitrile containing 0.1% trifluoroacetic acid (TFA). For analysis of individual beads, the selected beads were first suspended in minimum volume of acetonitrile and placed in a small petri dish to spread the beads apart and let the solvents evaporate. A small volume (1–2 µL) of matrix solution was then dispensed through a micropipette tip onto a bead; the solution, after a few seconds, was retrieved to the tip and spotted onto the MALDI target plate for analysis. For analysis during synthesis, as described above, a small sample (1–5 mg) was removed from synthesis, placed in a small column with frit, labeled with TMPP at the N-terminus, and deprotected. After gaseous degradation, the resin was suspended in minimum volume of 50% aqueous acetonitrile to extract the degraded peptides; the extract (1 µL) is then added to 10 µL matrix solution, and 1 µL of the mixture was then placed on the MALDI target plate for analysis. Additional information on sample preparation can be found in Supplementary information 3.

## Electronic supplementary material

supplemental info
