# Supplementary material for: Peptide Sequencing Directly on Solid Surfaces Using MALDI Mass Spectrometry
Source: Sci Rep. 2017 Dec 19;7:17811. doi: 10.1038/s41598-017-18105-3 (PMC5736625; doi:10.1038/s41598-017-18105-3)
Supplement: Supplementary file 1 — supplemental info [file 41598_2017_18105_MOESM1_ESM.pdf]

Peptide Sequencing Directly on Solid Surfaces Using MALDI Mass Spectrometry

Zhan-Gong Zhao*, Lalaine Anne Cordovez, Stephen Albert Johnston & Neal Woodbury*

Biodesign Institute Center for Innovations in Medicine, Arizona State University, Tempe, AZ 85281, United States of America

Supplementary Information

1, Solid phase peptide synthesis. Tenta Gel amino resin (EMD Millipore, Billerica, MA) was used in solid phase synthesis of all peptides reported here. The stepwise assembly of the peptide sequence was performed via addition of the corresponding Fmoc- or Boc-protected amino acids in presence of Diisopropylcarbodiimide (DIC) and 6-chloro-1-hydroxytriazole. Both Fmoc- and BOC-protected amino acids were from AAPPTEC (Louisville, KY). For peptide synthesis with Fmoc-protected amino acids, Fmoc was removed by treatment of peptide resin in 20% piperidine in DMF (5min +15min); after the synthesis, the side chain protection were removed by treatment of peptide resin in a solution of trifluoroacetic acid (TFA, 90%), water (2.5%), Ethanedithiol (2.5%), and triisopropylsilane (5%) for 3 hours. The resin was then washed with dichloromethane and Methanol, dried before use. In Boc-based synthesis, the BOC group was removed in 50% trifluoroacetic acid in dichloromethane (30min); the side chain protections of the peptide were removed by following a "low-high trifluoromethanesulfonic acid (TFMSA)" method: the dried resin (50mg) was first suspended in a low cleavage solution of m-Cresol (50uL), Dimethylsulfide (150uL), trifluoroacetic acid (250uL), Trifluoromethanesulfonic acid (TFMSA, 50uL), and 3,6-Dioxa-1,8-octane-dithiol (10uL) and shaken for 3 hours at 0-5oC; the resin, after the low cleavage solution was removed, was suspended in a high cleavage solution of trifluoroacetic acid (500uL), thioanisole (50uL), TFMSA (50uL), and 3,6-Dioxa-1,8-octane-dithiol (15uL) and shaken for 1.5 hours at room temperature. The resin was then washed with Dichloromethane, Methanol, and dried before use.

2, Ammonia gas system for peptide degradation. As shown in the following diagram, an ammonia gas chamber (3a) is linked to the ammonia gas cylinder (1) by an inlet tubing and an ammonia compatible gas pressure gouge (2), through which the pressure in the chamber can be adjusted. A valve (4) is placed at the

outlet to close the flow of ammonia gas and to maintain pressure in the chamber. At the end of an experiment, the valve is turned open to allow the ammonia gas in the chamber to be released through the outlet tubing and trapped in a water trap (6). The protection flask (5) is placed between the chamber and the water trap to prevent water from accidentally flowing back to the chamber. A prototype gas chamber (3b in the diagram) is made of stainless steel. At the bottom part of the chamber, seven cylindrical slots were machined to hold 50-mL Falcon tubes; 1x3 inch slides (9) can be placed in each tube for treatment. Therefore, seven slides of arrays can be treated in one experiment. Hundreds of individual resin beads fixed at the bottom of a petri dish (7), or tens of resin samples in small vials placed in a rack (8) can be placed in the upper space of the chamber for treatment. Based on throughput needed for an application, the chamber can be designed and machined in different shapes, sizes and with different materials. We thank Dr. Douglas Daniel for providing the following diagram.

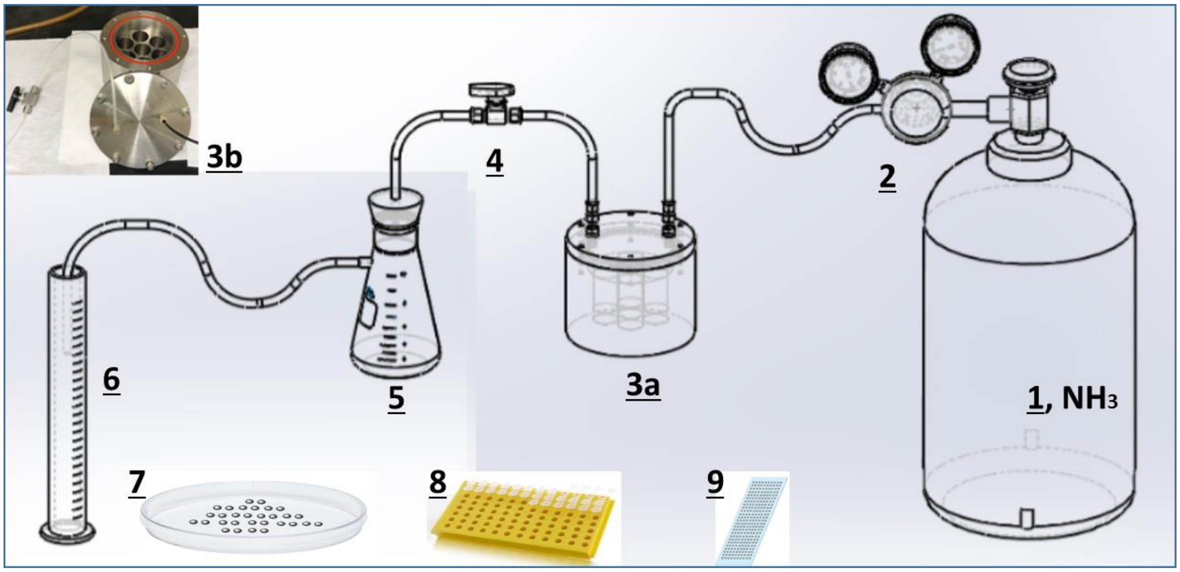

3, Sample preparation for MALDI MS analysis. Peptides on resin beads were analyzed either as individual beads or in small sample of resins (1-5mg) as described in method section. In each case, the quantity of peptide fragments depended largely on the loading of the resin and the extent of ammonia degradation and was mostly uncertain. For analysis of individual beads, as described in the method section, one microliter of matrix solution (CHCA saturated in 50%aqueous acetonitrile with 0.1%TFA) was added to individual beads before spotted onto the target plate for analysis. However, for analysis of

small sample of resins, we first added a minimum volume of 50% aqueous acetonitrile to the resin sample and extracted peptide into a solution. We then tested different mixing ratio of sample to matrix. Results for two conditions are shown in the following figure: a) one microliter of sample mixed with 10 microliter of matrix (CHCA saturated in 50% aqueous acetonitrile with 0.1% TFA); b) one microliter of sample mixed with one microliter of the same matrix solution before spotted on the target plate (MSP 96 polished steel target) for analysis. In most cases, the 1:10 mixing gave us the best results.

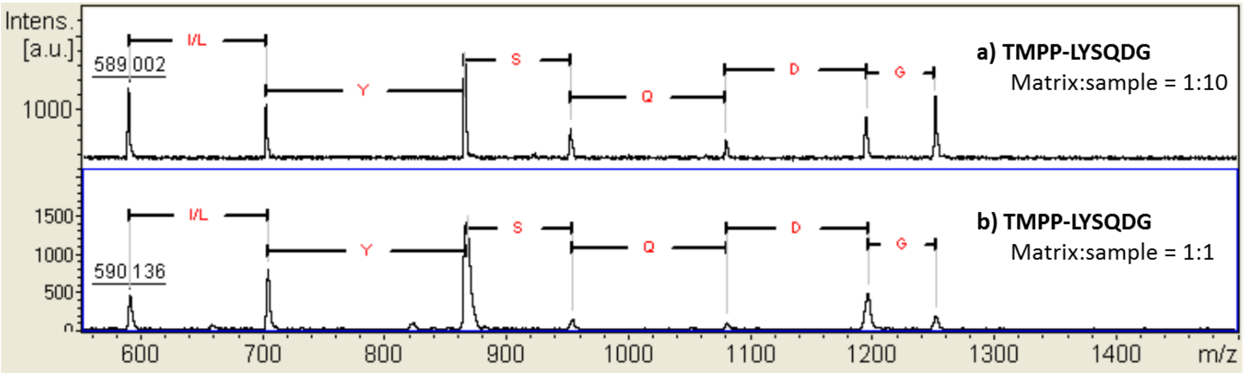

Although CHCA is well known as the matrix of choice for small peptides, we have tested the other two matrix compounds in our experiments. Three matric compounds, α-cyano-4-hydroxycinnamic acid (CHCA), 2,5-dihydroxybenzoic acid (DHB), and Sinapinic acid (SA), all in MALDI-MS grade, were purchased from Sigma. One microliter of sample was added to 10 microliter each of the following matrix solution: A) saturated solution of CHCA in 50% aqueous acetonitrile with 0.1% TFA; B) 20mg/mL of DHB in 30% acetonitrile in water with 0.1% TFA; C) saturated solution of SA in 30% acetonitrile in water with 0.1% TFA. Our results in the following figure showed that CHCA, as suggested by many others, is the most appropriate one for our purpose as the matrix.

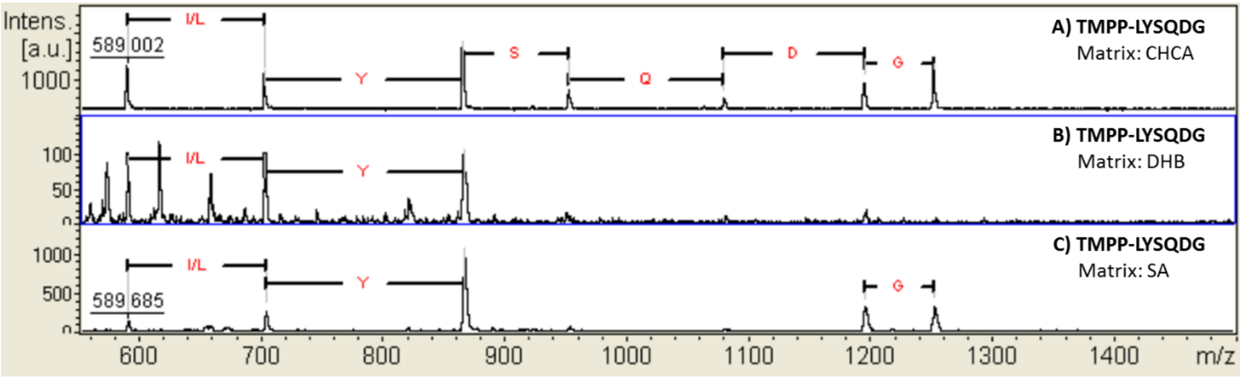

Supplementary Figures

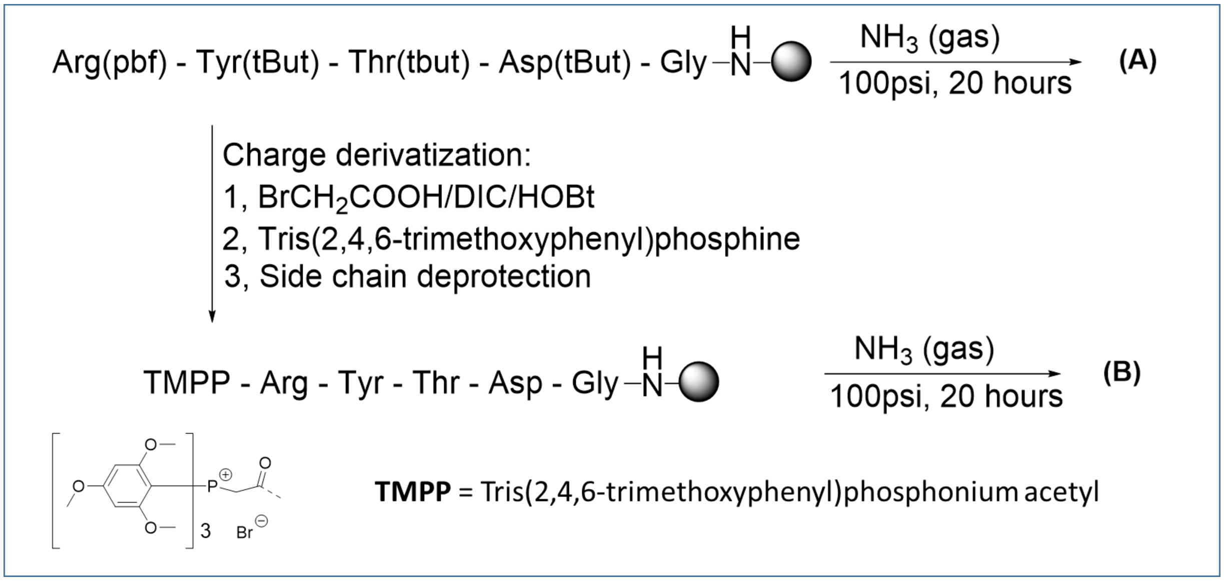

Supplementary Figure 1 | Peptide degradation in gas phase ammonia. (A), A 5-mer peptide with its side chain protection intact was treated in gaseous ammonia. (B), through a two-step process, the same peptide was first derivatized at its N-terminus using a group containing a fixed positive charge, TMPP. The side chain protecting groups were then removed. The labeled peptide was treated in gaseous ammonia. The ammonia treated samples (A) and (B) were analyzed by MALDI MS. The mass spectra are shown in **Supplementary Figure 2** and **Supplementary Figure 3**.

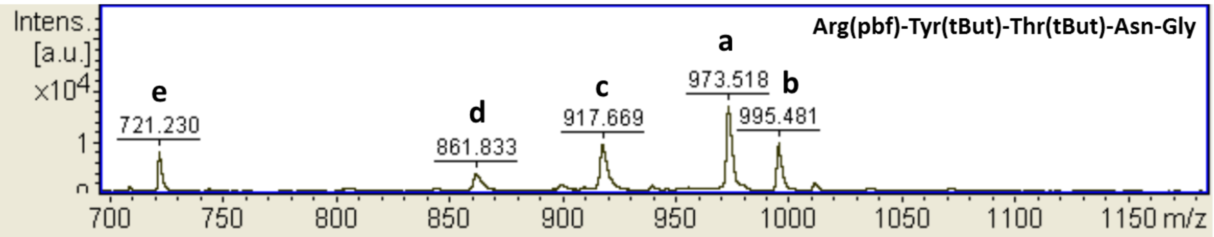

Supplementary Figure 2 | MALDI mass spectra of peptides after treatment in gaseous ammonia. The mass specta were not calibrated. Peaks labeled are fragments from protected peptide, Arg(pbf)-Tyr(tBut)-The(tBut)-Asn-Gly, a: M+H, b: M+Na, c: (M-tBut)+H, d: (M-2tBut)+H, e: (M-pbf)+H. All these molecular ions are corresponded to the full peptide with some side chain protecting groups removed during the process. No peptide bond cleavage were observed, indicating that they are quite stable in ammonia gas. Also note that the side chain protecting group of Asp(tBut) was removed during ammonia treatment and turned the amino acid to Asn.

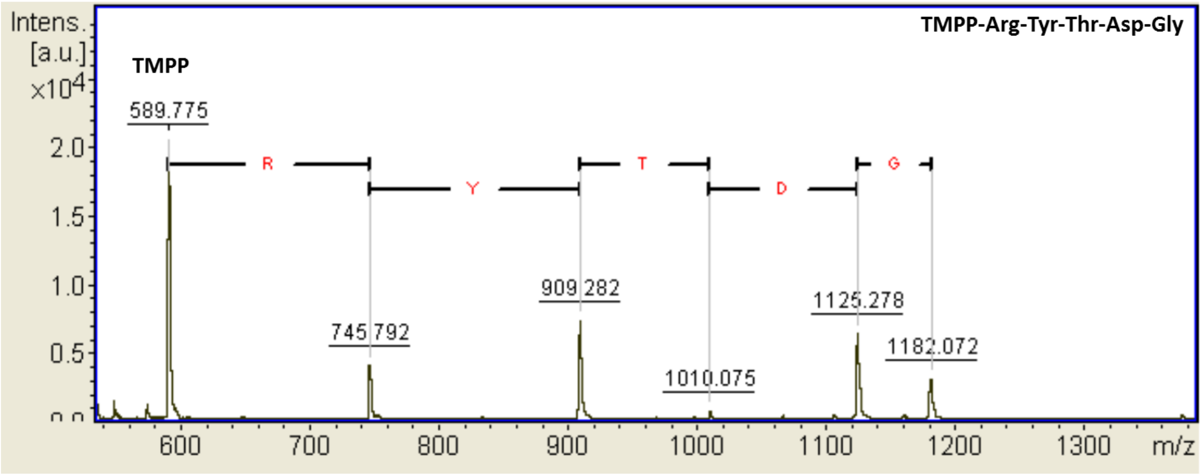

Supplementary Figure 3 | MALDI mass spectra of TMPP-labeled peptide after treatment in gaseous ammonia. The 5-peptide in supplementary Fig. 1 was labeled with TMPP using a two-step procedure. After the side chain protecting groups were removed, the labeled peptide was treated in ammonia gas before MALDI mass analysis. The mass spectrum was not calibrated. the spectrum is analyzed by using Bruker Daltonics flexAnalysis 3.0. The peptide sequence was correctly readout from the spectrum as TMPP-Arg-Tyr-Thr-Asp-Gly.

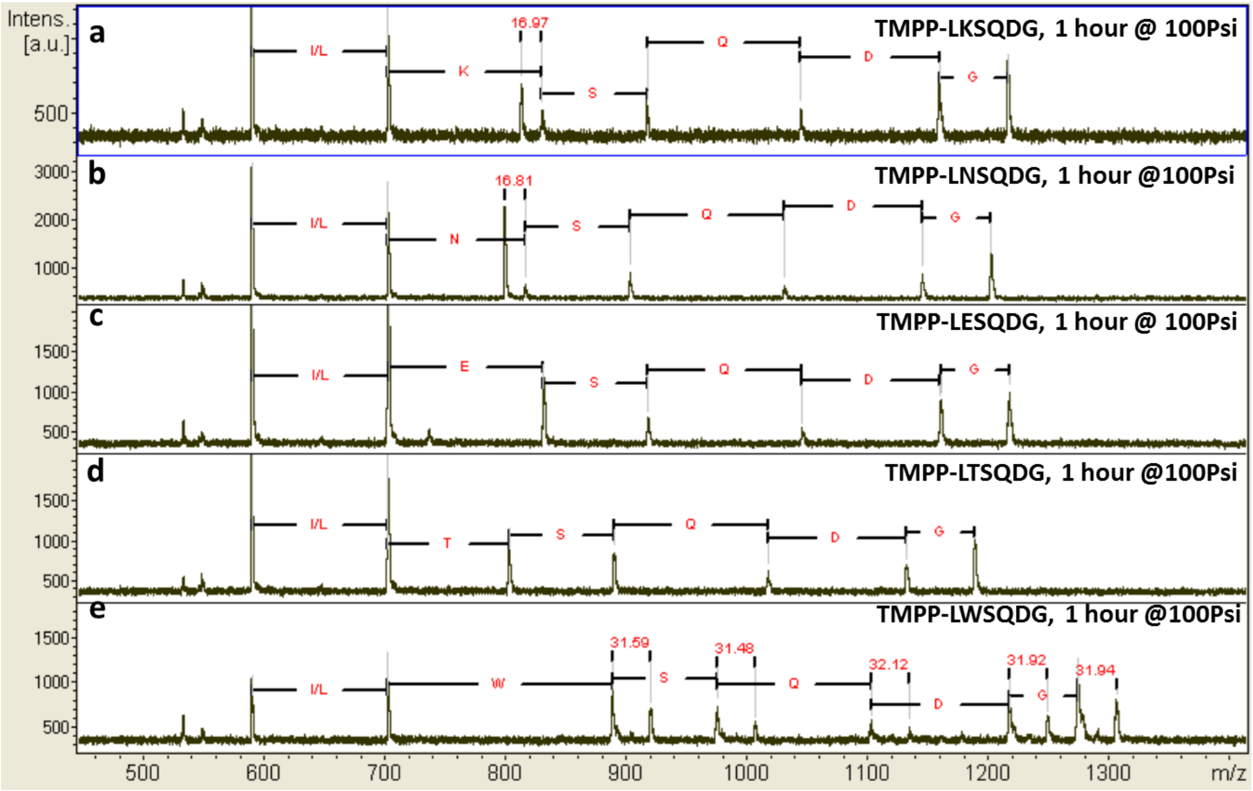

Supplementary Figure 4 | MALDI mass spectra of TMPP labeled six-mer peptides, TMPP-Leu-Xxx-Ser-Gln-Asp-Gle-TG. TG: tenta gel; (a) Xxx= Lys (K); (b) Xxx = Asn (N); (c) Xxx = Glu (E); (d) Xxx = Thr (T); (e) Xxx = Trp (W). This figure is the same as figure 4. The display range is reset to magnify the smaller peaks. The mass spectra were not calibrated.

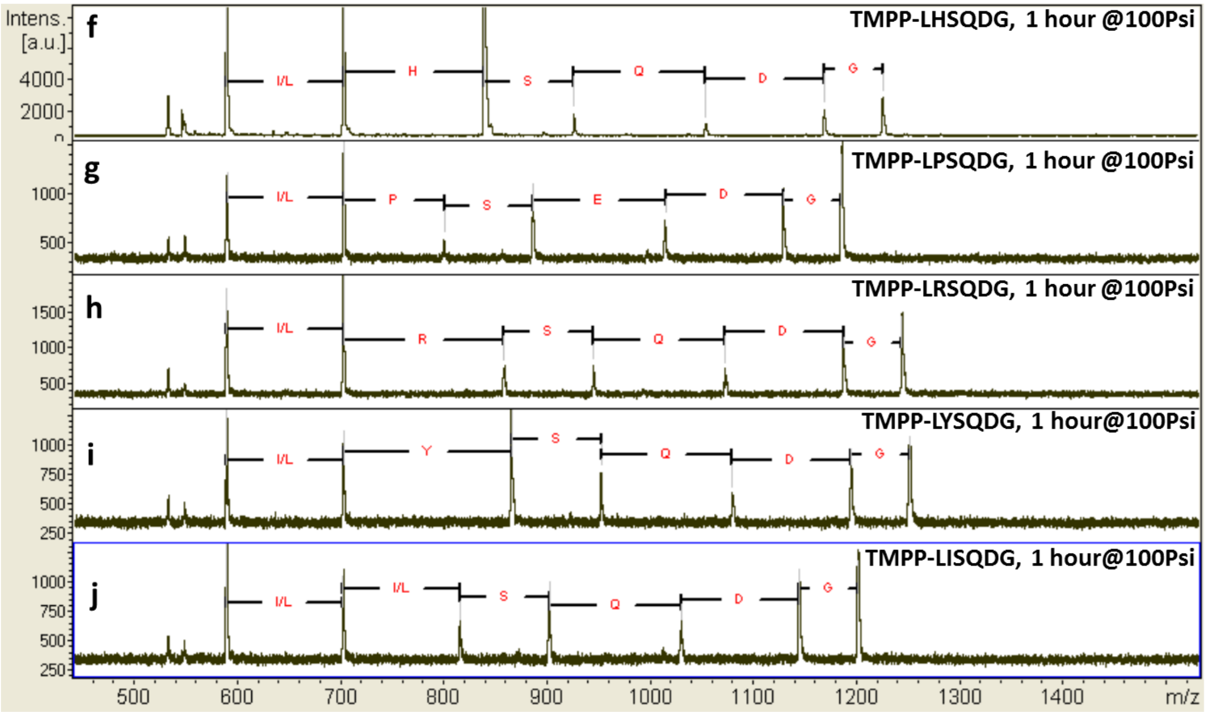

Supplementary Figure 5 | MALDI mass spectra of TMPP labeled six-mer peptides, TMPP-Leu-Xxx-Ser-Gln-Asp-Gle-TG. TG: = Tenta Gel; (f) Xxx= His (H); (g) Xxx = Pro (P); (h) Xxx = Arg (R); (i) Xxx = Try (Y); (j) Xxx = Ile (I). This figure is the same as Figure 5 (see in main text), the display range is reset magnify the smaller peaks. The mass spectra were not calibrated.

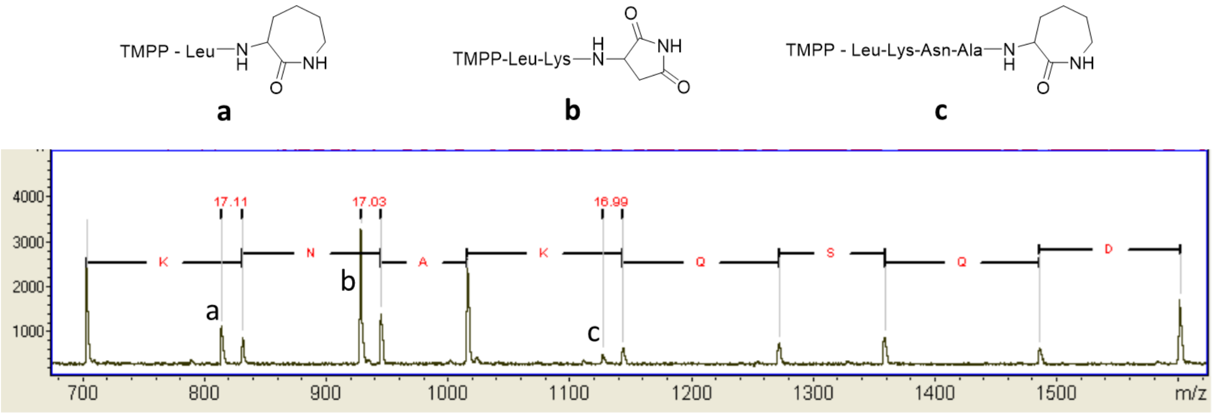

Supplementary Figure 6 | Deamination of Lysine and Asparagine in ammonia gas. Deamination (-17 Da) peaks are labeled as a for lys, b for Asn and c for Lys. No deamination is observed for the two Gln (Q) residues. This MALDI mass spectrum is part of Figure 6 in manuscript (display range is reset to magnify the smaller peaks in Figure 6). The spectrum was not calibrated.
